# Inpatient Rehabilitation Improves Physical and Mental Health in Multiple Myeloma: A Prospective Cohort Study

**DOI:** 10.3390/cancers18040588

**Published:** 2026-02-11

**Authors:** Jan Räder, Andriani Vasakou, Gabriele Ihorst, Sina Wenger, Georg Herget, Christine Greil, Ralph Wäsch, Monika Engelhardt

**Affiliations:** 1 Department of Medicine I (Hematology, Oncology and Stem Cell Transplantation), Medical Center—University of Freiburg, Faculty of Medicine, University of Freiburg, 79106 Freiburg, Germanyandivasakou@yahoo.gr (A.V.); sina.wenger@uniklinik-freiburg.de (S.W.); christine.greil@uniklinik-freiburg.de (C.G.); ralph.waesch@uniklinik-freiburg.de (R.W.); 2 Clinical Trials Unit, Medical Center—University of Freiburg, Faculty of Medicine, University of Freiburg, 79106 Freiburg, Germany; gabriele.ihorst@uniklinik-freiburg.de; 3 Department of Oral and Maxillofacial Surgery, Medical Center—University of Freiburg, Faculty of Medicine, University of Freiburg, 79106 Freiburg, Germany; 4 Department of Orthopedics and Trauma Surgery, Medical Center—University of Freiburg, Faculty of Medicine, University of Freiburg, 79106 Freiburg, Germany; georg.herget@uniklinik-freiburg.de

**Keywords:** rehabilitation, multiple myeloma, pre-post assessments, physical and mental health benefits

## Abstract

Introduction: This study evaluated the impact of standard inpatient rehabilitation on physical and mental health outcomes in patients with multiple myeloma (MM). Since prior data showed that physical activity (PA) is beneficial for patients with MM, this study assessed (1) the onset and (2) durability of these benefits with and without rehabilitation. Methods: Sixty patients with MM, undergoing a three-week rehabilitation program in the rehabilitation clinic of the University of Freiburg between April 2022 and September 2023, were assessed at three time points: baseline (T0), post-rehabilitation (T1), and 3-months post-rehabilitation (T2). Six patients, declining rehabilitation, were also examined. Tests included the timed-up-and-go-test (TUGT), handgrip strength, laboratory parameters, subjective fitness-rating, validated questionnaires for PA, fatigue, depression, and health-related quality of life (HRQoL: SF-12, R-MCI). Results: Patients showed meaningful improvements in physical function, TUGT, and grip strength from T0 to T1. Fatigue, depression, and HRQoL improved considerably. After their return home (T2), 80% of patients remained physically active. Patients reported substantially higher subjective physical fitness at T2 compared to T0, improving to 5.0 from 3.1 on a 10-point scale, respectively. Non-rehabilitation-undergoing patients were fitter at baseline but did not improve in any tests/questionnaires at T1. Conclusions: Structured three-week rehabilitation led to a substantial improvement in both the physical and psychological well-being of patients with MM, despite their compromised bone health. These effects persisted 3-months after patients’ dismission home. The non-rehabilitation group showed no comparable improvement, underscoring the potential benefit of structured rehabilitation in enhancing HRQoL, fatigue, and depression. Continued post-rehabilitation support is pertinent to sustain these benefits.

## Simple Summary

This study evaluated the effects of a standard 3-week inpatient rehabilitation program on physical and mental health outcomes in patients with multiple myeloma (MM). Sixty patients undergoing standard rehabilitation were assessed at baseline (T0), after rehabilitation (T1), and 3 months later (T2) and compared with six patients declining rehabilitation. Rehabilitation resulted in significant improvements in physical performance, muscle strength, fatigue, depression, and health-related quality of life. At T2, most patients remained physically active and reported higher subjective physical fitness than at baseline. In contrast, patients who did not undergo rehabilitation showed no meaningful improvements. These findings indicate that structured rehabilitation may provide sustained physical and psychological benefits for patients with MM. Continued post-discharge support should help to maintain these benefits.

## 1. Introduction

Physical activity (PA) has been shown to improve both physical functioning and mental well-being in cancer patients [1,2,3]. Common psychological comorbidities such as depression, anxiety, and reduced functional capacity frequently occur during cancer treatment but may improve over time with the clinical response and participation in supportive care programs [1,2,4].

Multiple myeloma (MM) is the second most common hematologic malignancy. Advances in treatment have led to substantial improvements in progression-free survival (PFS) and overall survival (OS); however, therapy should extend beyond disease control to include the prevention of adverse events (AEs) and preservation of health-related quality of life (HRQoL) [5,6,7].

The Revised Myeloma Comorbidity Index (R-MCI) is an MM-specific tool developed to assess overall health status and to support risk-adapted treatment decisions. It incorporates weighted prognostic factors identified through multivariate analysis, including impaired pulmonary and renal function, the Karnofsky Performance Status (KPS), frailty as defined by Fried criteria, age, and cytogenetic risk (http://www.myelomacomorbidityindex.org, accessed on 8 February 2026). Based on the R-MCI score, patients are classified as “fit” (R-MCI 0–3), “intermediate-fit” (R-MCI 4–6), or “frail” (R-MCI 7–9), categories that are associated with markedly different PFS, OS, and AE rates. As treatment responses are achieved and myeloma-related symptoms resolve, patients’ functional status, R-MCI, and HRQoL may improve [8,9].

In patients with cancer, PA has been shown to confer beneficial effects, with the strongest evidence derived from studies in breast and colorectal cancer [3,10,11]. Patients with hematological malignancies are also likely to benefit from exercise interventions; however, the available evidence remains limited [12,13]. In MM specifically, data from randomized controlled trials (RCTs) are scarce. However, patients may profit from physical activity to better counteract physical deconditioning, prevent sarcopenia, and ameliorate fatigue [14,15,16], particularly as contemporary MM treatment has become more intensive and continuous rather than time-limited [5,17,18]. Although preliminary studies support the feasibility and HRQoL benefits of exercise during therapy [19], PA is not a standard component of MM care. Nevertheless, rehabilitation programs are aiming to integrate individualized training into routine practice [20].

Our prior analysis comparing physically active and inactive patients with MM indicated a better response, PFS, OS, R-MCI, and QoL in active patients [2]. In the following RCT, we compared patients trained to achieve World Health Organization (WHO)-compliant PA [1] to patients with activity as usual. According to WHO recommendations, cancer patients should complete at least 150 min of moderate endurance training per week and perform at least two training sessions to specifically strengthen large muscle groups [1,21]. In the RCT of WHO-trained vs. control patients with MM, we analyzed demographics, comorbidities, induction tolerance, treatment responses, the Timed-up-and-go-test (TUGT)-, R-MCI-, HRQoL via the Short Form-12 Health Survey (SF-12), biomarkers and event-free survival (EFS) [1]. Training endurance was tracked via smartwatches and diaries. Notable improvements were achieved in trained MM vs. control patients [1], suggesting that rehabilitation programs, routinely offered after intensive MM therapies [5], may induce similar benefits.

Thus, given that many patients with MM are undergoing rehabilitation following intensive therapies and that individualized, moderate training programs are a core component of rehabilitation, we aimed to evaluate the effects of a structured rehabilitation program [1,2,22]. Building on previous findings that demonstrated the safety and efficacy of PA in patients with MM [1,2,22], this study assessed whether rehabilitation leads to measurable improvements in functional parameters. Specifically, we sought to determine (1) how quickly physical and mental improvements occur and (2) how sustained these benefits are over time. Evaluations were conducted at three time points: the beginning of rehabilitation (T0), after three weeks upon completion of the program (T1), and three months post-rehabilitation (T2), when patients had resumed their daily routines and ideally maintained aspects of the rehabilitation program at home.

## 2. Material and Methods

### 2.1. Participants

This exploratory prospective study investigated patients with MM undergoing a standard three-week rehabilitation program at the Tumorbiology Rehabilitation Clinic Freiburg. An 18-month recruitment period was chosen to align with sample sizes reported in comparable studies [23,24,25,26]. Eligible participants were adults (≥18 years) with confirmed MM capable of providing informed consent and completing assessments. Exclusion criteria included acute medical instability or other conditions precluding functional testing or data collection. Patients were consecutively enrolled.

The study was conducted according to the Declaration of Helsinki and Good Clinical Practice. Ethical approval was obtained from the independent ethics committee (EV #20-1351 and 173/20), and all participants provided written informed consent.

### 2.2. Study Design and Outcome Measures

Primary outcomes included changes in functional performance and HRQoL at baseline (T0), after rehabilitation (T1), and three months post-rehabilitation, after patients had returned home (T2).

Functional performance was assessed via the TUGT and grip strength. HRQoL was measured with the SF-12 questionnaire. Depression was evaluated via HDS-17 and fatigue using Common Terminology Criteria for Adverse Events (CTCAE) of the National Cancer Institute (NCI), as described [1,22]. Secondary outcomes included self-reported PA levels, perceived fitness, exercise motivation, engagement in structured PA, and laboratory biomarkers. Socioeconomic factors were recorded to explore potential barriers to care, as performed previously [1,2]. The last follow-up (T2) was conducted via mailed test and questionnaire forms and included questionnaire-based instruments.

### 2.3. Assessment

Interviews and functional testing were performed by a single trained physician (AV) to ensure consistency throughout the study. Baseline clinical data, including disease and treatment history, were extracted from medical records, as summarized in Table 1. Remission was classified according to the International Myeloma Working Group (IMWG) criteria. Functional performance was assessed via validated TUGT and grip strength protocols (Table 2) [1]. Laboratory biomarkers were measured according to prior studies [1,2,27,28] and included hemoglobin, albumin, pro-brain natriuretic peptide (proBNP), C-reactive protein (CRP), high density lipoprotein (HDL), and lactate dehydrogenase (LDH; Table 3). Depression, fatigue, HRQoL changes via R-MCI and the SF-12-questionaire [29], muscle strengthening, and PA were explored at T0, T1, and T2 (Table 4). Depression (HDS-17) and anemia and fatigue (CTCAE of NCI grade ≥2) were evaluated. PA behavior, subjective fitness, and exercise motivation were recorded using a validated PA questionnaire as described [1,22].

Patients were considered physically active if they engaged in at least one hour of moderate intensity exercise per week [22]. Compliance with the WHO PA guidelines [21] was not an intended goal of this study.

### 2.4. Statistical Analysis

Data were analyzed using SAS statistical software version 9.4 (SAS Institute Inc, Cary, NC, USA). Since this was an exploratory trial with the primary aim to assess feasibility and a first evaluation of efficacy and safety, no formal sample size calculation was performed. Descriptive analyses were conducted using the median (range) for continuous variables and reporting Wilcoxon’s signed rank test for pre-post comparisons. Since deviations from normality were frequently observed, non-parametric methods were applied throughout the analyses to ensure the robustness of the results. For categorical variables, absolute and relative frequencies were calculated, and the pre–post comparisons were performed with McNemar’s test. As a measure of effect, we report absolute and relative frequencies of patients who improved, e.g., from ‘depression’ to ‘no depression’, or from ‘no PA’ to ‘PA’; thus, frequencies of patients with deterioration can then be derived. We considered the reported *p*-values as descriptive information; therefore, *p*-values are reported in tables and figures but not discussed in the text.

## 3. Results

### 3.1. Rehabilitation Program

The rehabilitation program aimed to improve the physical function, psychological well-being, and participation in daily activities and was individualized and multimodal. Patients received approximately 15–30 h of therapy per week, including strength and endurance training, isometric core stability exercises, psychosocial support, counseling, and side-effect management. PA sessions typically lasted 30–60 min each, with 4–6 training and support sessions, tailored to each patient’s functional capacity. Examples for PA sessions included guided walking groups offered 3 times per week for ½ or 1 h, yoga, pilates and/or meditation groups, and machine-based or bodyweight-based muscle strengthening sessions. Patients could attend cooking groups, art, music, and recreation offers and received dietary consultation and psychosocial interventions. Osteolytic patients underwent orthopedic evaluation and received specific risk counseling based on their bone status and stability for exercises/gymnastics. Patients were reassessed on a weekly basis. The number of intervention offers ranged from 4 to 6/day for each patient, based on interest, fitness, and eagerness. An interdisciplinary team was key to provide individualized rehabilitation offers to patients.

### 3.2. Study Accrual

**Figure 1:**
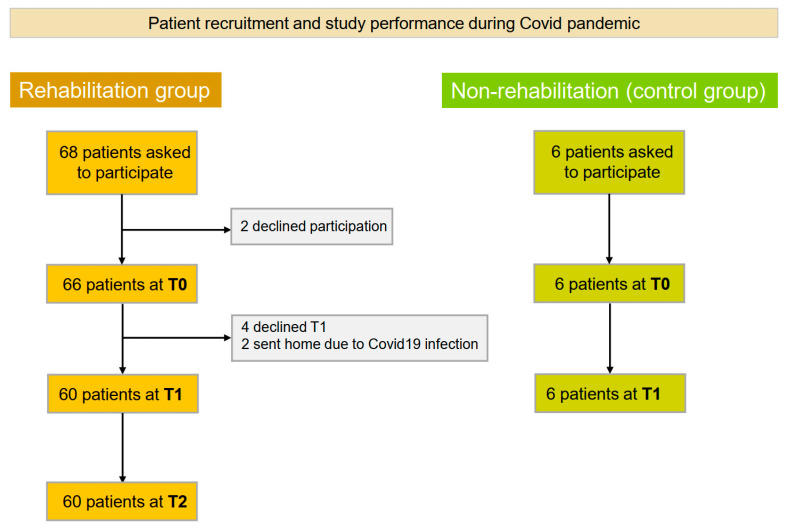
Consort diagram: patient recruitment and study performance during the prolonged COVID pandemic. Definitions + abbreviations: T0: baseline; T1: upon completion of rehabilitation; T2: 3 months post rehabilitation, after patients had returned home.

The study was performed during the prolonged COVID pandemic with the need for individual 1:1 assessments between the patient and examiner (AV) due to contact restrictions and hygiene regulations. As a result, enrolment was halted after inviting 68 patients with MM to participate, with 66 consenting. Two patients discontinued rehabilitation due to severe COVID-19 infection. Another four patients unfortunately declined the T1 follow-up, leading to their exclusion, since T1 and T2 data could not be acquired. In cases of delayed responses or incomplete questionnaires at T2, the study physician (AV) contacted patients to ensure optimal data collection, which proved to be successful, with full data acquisition of all 60 patients for T0, T1, and T2 (Figure 1).

### 3.3. Patient Characteristics

At the initial diagnosis (ID) of MM, the median patient age was 59 years (range: 32–71), and at the time of rehabilitation, it was 63 years (Table 1). Patient characteristics were representative of typical referral centers, with a near-equal gender distribution (53% male, 47% female). Most patients were married (75%), and half (50%) were still working, reflecting the age demographics. The majority reported a sufficient income, in line with prior analyses [1,2]. Nearly all patients were Caucasian (92%), in line with the regional demographics of the Tumorbiology Rehabilitation Clinic.

Most participants (90%) had typical MM, while few (10%) had plasmacytoma, AL-amyloidosis, or SMM. Paraprotein types of heavy and light chain types were as expected, with advanced ISS and R-ISS stages 2/3 present in 67% and 80%, respectively. Three or more osteolytic lesions were common (67%), whereas fewer (0–2) osteolytic lesions were less frequent (33%).

Regarding treatment status, 82% were in complete remission (CR) or very good partial remission (VGPR), while 18% were in partial remission (PR). The majority (87%) had undergone autologous stem cell transplantation (ASCT; Table 1). Most patients were attending rehabilitation for the first time (72%), while 28% were undergoing a second or subsequent rehabilitation. Patient characteristics of the four patients turning down the T1 + T2 assessments did not differ from the rest of the cohort.

### 3.4. Physical Activity, Biomarkers, and Impact of Rehabilitation on the Physical Performance

Objective measures of physical performance aligned with subjective improvements. Median TUGT time improved from 11.3 s at T0 to 10.0 s at T1, indicating enhanced mobility. In line, grip strength improved from T0 to T1, with a considerable median increase of 2 kg in the right hand and 3 kg in the left hand. Thus, in line to the TUGT, grip strength was not substantially impaired compared to the age-adapted norm. The body mass index (BMI) remained stable at 26 kg/m^2^ across T0 and T1, but the T0-range was higher (Table 2).

Expectedly, laboratory biomarkers remained largely unchanged during the three-week rehabilitation (Table 3). Notable exceptions were reductions in the median HDL (55 → 51 mg/dL) and LDH (203 → 191.5 U/L; Table 3), likely related to dietary improvements and hematological recovery during the rehabilitation stay, in line with prior analyses [2,27,30,31].

**Figure 2:**
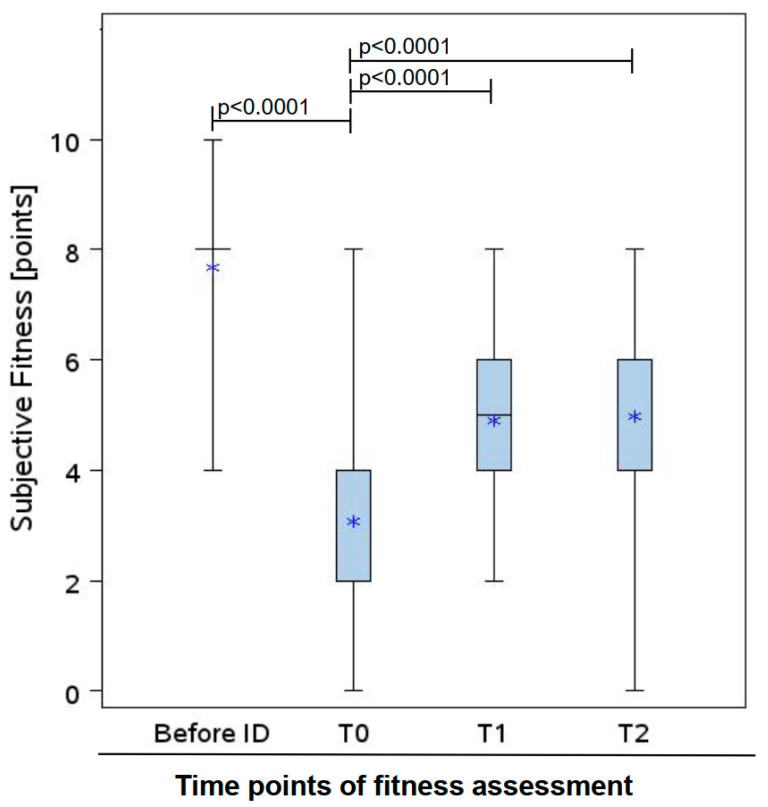
Mean self-rated physical fitness of rehabilitation MM cohort at different time points: before initial diagnosis (ID) of MM, at T0, T1, and T2, with 95% confidence intervals (n = 60). Self-rated physical fitness was scored on a normalized 10-point scale (10 points = best possible fitness, 0 points = worst possible fitness). Boxes display the interquartile range (lower quartile–upper quartile), whiskers display the range (minimum–maximum). The median is represented by a bar and the mean by a star. Before ID of MM, the upper and lower quartile at ID are equally high (score 8); thus, no box is displayed. Definitions + abbreviations: ID: initial diagnosis, T0: baseline; T1: upon completion of rehabilitation; T2: 3 months post rehabilitation.

Self-rated physical fitness, scored on a normalized 10-point scale (10 points = best possible fitness, 0 points = worst fitness), declined substantially from pre-MM diagnosis levels (7.7) to 3.1 at T0. At T1, it increased to 4.9 and was maintained at 5.0 at T2 (Figure 2).

Table 4 provides a detailed overview of PA and muscle-strengthening engagement before and after rehabilitation. Prior to the ID of MM, 47/60 of patients (78%) had exercised regularly. Participation in PA and physiotherapy was strongly encouraged during the Tumorbiology rehabilitation, resulting in a marked increase in PA adherence from 33% (20/60) at T0 to 98% (59/60) at T1. At T2, 80% (48/60) of patients continued to engage in PA, suggesting sustained behavioral changes. Likewise, the proportion of patients performing muscle-strengthening exercises increased notably from 22% (13/60) at T0 to 48% (29/60) at T2, indicating greater uptake of resistance training due to rehabilitation.

Fatigue improved from T0 to T1, and depression substantially declined from 38% at T0 to 8% at T1 and increased again at T2 (18%).

HRQoL via the SF-12 questionnaire in terms of PCS and MCS improved from T0 to T1, from a median of 36 to 42 and 51 to 56, respectively. At T2, both declined slightly (Table 4). At T0 and T2, 82% of patients scored below the general population’s mean PCS, but this temporarily improved at T1. The proportion of patients scoring above average increased from 12% at T0 to 17% at T2, suggesting some persistent benefit for a subset of patients.

While 53% of patients reported above-average MCS scores at T0, this proportion increased to 80% at T1 and remained at 58% at T2. The proportion of patients with below-average MCS scores decreased from 45% at T0 to 40% at T2. During the three-week rehabilitation, the R-MCI of fit and intermediate-fit patients did not change substantially (Table 4).

### 3.5. Comparison of Outcome Parameters with the Non-Rehabilitation (Control) Group

Six patients with MM who did not undergo inpatient rehabilitation were evaluated to explore potential differences in outcomes to the rehabilitation cohort (Table S1). All had undergone ASCT and were evaluated at two time points: four weeks post-ASCT (analogous to T0) and again three weeks later (analogous to T1). Reasons for non-participation in rehabilitation included preference for home recovery (in all 6), plus an intention to return to work (n = 3), perceived sufficient fitness (n = 2), and orthopedic limitations due to osteolytic lesions, which did not allow rehabilitation exercises (n = 1; multiple reasons possible).

As shown in comparison to the rehabilitation group in Table S1, both groups were largely comparable, although the non-rehabilitation group was older with a median of 69 years (range: 60–75). All non-rehabilitation patients were in CR after ASCT. Laboratory profiles were similar.

Of interest, the non-rehabilitation group estimated their fitness at T0 (7.0 of 10 points) and T1 (improved to 7.7) better than the rehabilitation group (T0: 3.1 → T1: 4.9), and 0% had depression via HDS-17. However, the number of fit patients via R-MCI did not increase, nor did SF-12-QoL-, fatigue-, PA-, and muscle-strengthening-results show improvements (Table S1).

### 3.6. Adverse Events and Safety

No AEs related to PA or other rehabilitation interventions were observed. Since the study was performed during the prolonged COVID-19 pandemic, two patients had to discontinue rehabilitation due to severe COVID-19 infection.

### 3.7. Time Requirements for Assessments

The time investment for assessments was recorded across all time points. At T0, interviews and physical testing for the rehabilitation group required a median of 33 min (range: 22–57). T1 required a median of 25 min (range: 18–50). The T2 follow-up, conducted via mail with validated questionnaires, required a median of 22 min (range: 20–26) per respondent. The interview duration for the non-rehabilitation (control) group was shortest with a median time of 18 min (15–25) for T0 and T1.

## 4. Discussion

This exploratory prospective cohort study evaluated the short- and mid-term outcomes of a standard structured three-week inpatient rehabilitation program in patients with MM, primarily following ASCT. Given that patients with cancer, including those with MM, often participate in rehabilitation programs, where moderately intensive and personalized exercise is a core intervention, we aimed to assess their impact—building on previous findings from our group [1,2,22].

All study participants received rehabilitation at a specialized MM-focused facility. Over a three-week period, patients showed clinically relevant improvements in functional performance, particularly in the TUGT and handgrip strength, reflecting gains in mobility and muscular function. These findings are consistent with prior research highlighting the feasibility and efficacy of physical rehabilitation in cancer patients. Cenik et al. found that 50% of patients with MM achieved the performance values of healthy individuals in the TUGT and grip strength [32], and while earlier studies were unable to demonstrate substantial improvements through training interventions [33], others have [1,3,17,34,35,36]. Our findings extend this evidence by showing that even a relatively short three week rehabilitation can yield measurable physical benefits, despite the challenges posed by MM-related bone disease and exposure to intensive chemotherapy. Although fatigue, pain, and disease-related anxiety can limit PA in patients with MM, up to 75% reported a desire to increase their activity levels [1,2,22,37]. In our cohort, patients did not only improve physically but also experienced a pronounced reduction in fatigue and depressive symptoms, alongside HRQoL improvements. These psychological benefits were likely multifactorial, driven by physical improvements, structured daily routines, social interactions, and supportive therapeutic environments.

In line, the mental health condition can improve with PA, particularly depression, which is more prevalent in physically inactive individuals [38,39]. Prior research has linked inactivity in patients with MM to worsened mental and emotional well-being [2,31,38,40]. The beneficial effects of exercise on depressive symptoms are established [35,36]. Accordingly, structured PA has been successfully implemented as an essential component of multidisciplinary treatment approaches for depressive patients [41] and is a vital part of rehabilitation programs. A multicenter study by Jordan et al. demonstrated a causal link among depression, fatigue, and QoL in patients with MM who had inadequate levels of PA [7]. Other studies have confirmed that PA can significantly improve the QoL of cancer patients, including those with MM [1,2,31,42].

Of note, despite clear short-term benefits in HRQoL, TUGT, muscle strength, fatigue, and depression, the R-MCI improved less [35]. The proportion of fit and intermediate-fit patients shifted only slightly from 52%/48% at T0 to 53%/47% at T1, respectively. To better capture patients’ gains in physical performance, therapy tolerance, and HRQoL, a latency of approximately one year appears more suitable than three months or less [1]. Likewise, the common practice of repeatedly assessing HRQoL domains (i.e., in clinical studies) over short time periods may be less informative in light of our findings [1,8].

Although our non-rehabilitation comparison group was small and non-randomized, interesting contrasts emerged: these patients were older (median 69 years), 50% still working, similarly affected by multiple bone lesions (67%) and with comparable laboratory parameters at T0 (Table S1). They reported higher self-assessed fitness and showed no depression, although fatigue remained high at 67% at both T0 and T1. Different from the rehabilitation group, they showed no improvements in fatigue, depression, or HRQoL at T1 (Table S1). While the interpretation is limited by the sample size and potential selection bias, the findings suggest that structured rehabilitation may offer specific psychological and QoL benefits that are not attainable to the extent of rehabilitation programs through unsupervised recovery alone [35]. While this prospective cohort study cannot provide the level of evidence of a large RCT, it serves as a basis for future investigations. Statistically meaningful conclusions will require a larger non-rehabilitation control group in subsequent studies.

Our study provides valuable insights into the benefits of rehabilitation, particularly improvements in PA, fatigue, and mental health. Key strengths include the comprehensive assessment strategy (combining objective performance tests, self-reported outcomes, and clinical parameters), uniform data collection by a single independent physician, and complete follow-up for all 60 participants for the three time points, T0, T1, and T2. The independence of the interviewer, unaffiliated with the rehabilitation clinic, the single, repeatedly awarded rehabilitation clinic (1. prize award winning rehabilitation clinic of Germany) for this study, with an outstanding cancer rehabilitation program and uniform MM cohort, likely fostered candid responses, reducing social desirability bias. Although exercise intensity and adherence may have varied due to the individualized rehabilitation approach, this variability reflects real-world rehabilitation practice and therefore represents a deliberate and important strength of the study design. A more rigid standardization of exercise exposure would have conflicted with the clinical principle of individualized rehabilitation and may have reduced the external validity of our findings.

Our study can be criticized for its retrospective assessments of pre-rehabilitation fitness that are also prone to recall bias, and thus, its needs confirmation in subsequent analyses. Data collection at T2 was performed via mail, introducing possible bias compared to in-person assessments, and lacked data on fatigue, R-MCI, TUGT, and grip strength. The collection of these data would have required patients to return to the rehabilitation clinic 3 months after the completion of rehabilitation, which would have posed immense time expenditure and costs and thus was impossible to perform. Between T1 and T2, patients resumed their daily lives at home, and lifestyle-related confounding factors may have affected the results. Whether these changes were independent of or induced by rehabilitation cannot be determined from the present data and will require larger follow-up studies. Conducting the study during the extended course of the COVID-19 pandemic proved challenging due to substantial pandemic-related restrictions. Some patients originally scheduled to participate contracted COVID-19 at the beginning of their rehabilitation stay and were unable to continue participation.

## 5. Conclusions

This prospective exploratory study demonstrated that structured inpatient rehabilitation following ASCT is safe and effective in improving physical performance, mental health, and HRQoL in patients with MM. Considerable gains were observed in objective measures such as grip strength and TUGT, alongside improvements in patient-reported outcomes, including self-rated physical fitness, SF-12, and HDS-17 scores. Although some decline was observed at the three-month follow-up (T2), most improvements, especially in mental well-being, persisted beyond the immediate rehabilitation phase. Importantly, no AEs related to PA or therapeutic interventions were observed, underscoring the safety of rehabilitation programs, even in a population recovering from intensive oncological treatment. Our findings highlight the value of timely and targeted rehabilitation in MM and cancer care. They also underscore the need for structured post-rehabilitation support to maintain PA and preserve QoL gains over time. Establishing outpatient follow-up strategies and integrating digital exercise support tools may help to mitigate the observed decline after discharge. Future studies should investigate these approaches in larger, controlled cohorts. Our MM self-advocacy group (https://www.myelom-südwest.de/index.php, accessed on 8 February 2026) actively supported the implementation of this study and is engaged in sharing its findings. Their participation underscores the relevance of this study and reflects the growing interest among various cancer individuals, including those living with MM, in comprehensive survivorship care [35,36].

## Figures and Tables

**Table 1:** Patient characteristics of rehabilitation-undergoing MM patients (n = 60).

Variables	Median (Range)/n (%)
Age at initial diagnosis [years]	59 (32–71)
Age at T0 [years]	63 (37–74)
Gender: male/female	32 (53)/28 (47)
Ethnicity: Caucasian/others	55 (92)/5 (8)
Marital status: married/others	45 (75)/15 (25)
Employment: retired/still working	30 (50)/30 (50)
Income: medium or high/low *	59 (98)/1 (2)
MM/plasmacytoma, AL-amyloidosis or SMM	54 (90)/6 (10)
Type of MM: IgG/IgA/LC/IgM and others	44 (74)/11 (18)/2 (3)/3 (5)
LC type: kappa/lambda/biclonal	41 (68)/18 (30)/1 (2)
ISS: I/II/III	20 (33)/26 (43)/14 (24)
R-ISS: I/II/III	12 (20)/35 (58)/13 (22)
Osteolytic lesions: 0–2/3 or more	20 (33)/40 (67)
Current state of disease: nCR/CR/vgPR/PR	1 (2)/26 (43)/22 (37)/11 (18)
Induction anti-MM treatment: Daratumumab-VCD/VCD/other	10 (17)/48 (80)/2 (3)
Stem cell transplantation performed: ASCT/ASCT + allo-SCT/standard therapy	52 (87)/5 (8)/3 (5)

Definitions + abbreviations: T0: time point zero, baseline; MM: multiple myeloma; n: number; AL-amyloidosis: amyloid light chain; SMM: smoldering multiple myeloma; LC type: light chain type; ISS: international staging system; R-ISS: revised international staging system; CR: complete remission, nCR: almost complete remission, vgPR: very good partial response, PR: partial remission. VCD: bortezomib, cyclophosphamide and dexamethasone. Other: Daratumumab alone + immunoglobulines. ASCT: autologous stem cell transplantation, allo-SCT: allogeneic stem cell transplantation. * Income: medium-high > 1100 €/month, low < 1100 €/month. White background: sociodemographic characteristics, light grey background: disease characteristics, dark grey background: remission state and treatment.

**Table 2:** Objective physical fitness and body constitution of patients.

Variables	T0 Median (Range)	T1 Median (Range)	T0 → T1 Difference Median (Range)	*T0* *→ T1* *p-Value*
TUGT [seconds]	11.3 (7.5–25.0)	10.0 (7.3–24.0)	−0.6 (−5.4–1.5)	*<0.0001*
Grip strength right [kg]	25 (5–53)	27 (5–55)	2.0 (−4.0–15.0)	*<0.0001*
Grip strength left [kg]	24 (5–45)	27 (6–47)	2.0 (−7–14.0)	*<0.0001*
BMI [kg/m^2^]	26 (18–44)	26 (18–43)	0 (−1–1)	*1*

*p*-values calculated using the Wilcoxon signed-rank test. Definitions + abbreviations: TUGT: Timed-up-and-go-test, kg: kilogram, m^2^: square meter; BMI: body mass index.

**Table 3:** Biomarker analysis at the start and end of rehabilitation (n = 60).

Variables	T0 Median (Range)	T1 Median (Range)	T0 → T1 Difference Median (Range)	*T0* → *T1* *p-Value*
Hemoglobin [g/L]	12.5 (8.4–15.2)	12.8 (8.0–15.9)	0.1 (−5.0–1.3)	*0.1454*
Albumin [g/dL]	4.35 (3.1–5.0)	4.4 (3.7–5.1)	0 (−0.7–0.7)	*0.4955*
proBNP [pg/mL]	111.5 (50–1473)	120 (50–1473)	0 (−724–194)	*0.2249*
CRP [mg/L]	3 (1.5–58)	3 (3–83)	0 (−51.7–77.6)	*0.3500*
HDL [mg/dL]	55 (30–106)	51 (22–111)	−2.5 (−32–13)	*0.0008*
LDH [mg/dL]	203 (141–390)	191.5 (133–349)	−8 (−95–100)	*0.0004*

*p*-values calculated using the Wilcoxon signed-rank test. Definitions + abbreviations: n: number; proBNP: pro-brain natriuretic peptide; CRP: C-reactive protein; HDL: high-density lipoprotein; LDH: lactate-dehydrogenase.

**Table 4:** Physical activity, fatigue, depression, HRQoL, and R-MCI changes at T0, T1, and T2 time points.

Variables		T0 [n (%)]	T1 [n (%)]	T2 [n (%)]	Improved T0 → T1 [n (%)], *p*-Value	Improved T0 → T2 [n (%)], *p*-Value
PA	Performance of PA (yes/no) *	20 (33)/40 (67)	59 (98)/1 (2)	48 (80) **/12 (20)	39 (65), <0.0001	29 (48), <0.0001
	Muscle strengthening (yes/no)	13 (22)/47 (78)	- ***	29 (48)/31 (52)		21 (35), 0.0017
Fatigue ^1^	Mild/moderate	49 (82)/11 (12)	58 (96)/2 (4)	- ****	9 (15), 0.0027	- **
Depression ^2^	Yes/no	23 (38)/37 (62)	5 (8)/55 (92)	11 (18)/49 (82)	18 (30), <0.001	16 (27), 0.0073
R-MCI	Fit/intermediate-fit	31 (52)/29 (48)	32 (53)/28 (47)	- ****	2 (3), 0.56	
		**T0**	**T1**	**T2**	**T0 → T1**	**T0 → T2**
HRQoL PCS	Median (range): PCS score	36 (11–62)	42 (20–57)	37 (17–56)	4 (−11–27), <0.0001	1 (−16–28), 0.15
	n (%): better/average/worse ^3^	7 (12)/4 (7)/49 (82)	14 (23)/0 (0)/46 (77)	10 (17)/1 (2)/49 (82)		
HRQoL MCS	Median (range): MCS score	51 (19–65)	56 (35–67)	53 (24–68)	7.5 (−24–36), <0.0001	3.5 (−17–29), 0.023
	n (%): better/average/worse ^3^	32 (53)/1 (2)/27 (45)	48 (80)/3 (5)/9 (15)	35 (58)/1 (2)/24 (40)		

*p*-values calculated using the McNemar or the Wilcoxon signed-rank test. Definitions + abbreviations: **n: number;** PA: physical activity; HRQoL: health-related quality of life; PCS: physical component summary; MCS: mental component summary; R-MCI: revised myeloma comorbidity index. 1: Fatigue assessed according to CTCAE; 2: depression according to Hamilton (HDS-17); 3: MCS and PCS scores range from 0 to 100, with 50 representing the average score in the general population. “Better” equals a score above 50, while “worse” depicts a score value of 49 or lower. * According to BORG rating of perceived exertion scale: at least moderate exertion, 11 or above. ** 52 (87%) planned to participate in further exercise interventions when asked at T1. *** Not assessed at T1, because muscle strengthening was an essential part of the Tumorbiology Rehabilitation program and performed by all 60 MM patients, whereas 3 months after rehabilitation (T2), the non-guided persistence and improvement to T0 was inquired. **** Fatigue and R-MCI were not assessed 3 months after rehabilitation (T2) because confirmation by the examiner (AV) was unfeasible, due to patients being dismissed home.

## Data Availability

The data that support the findings of this RCT are available from the corresponding author upon reasonable request.

## Supplementary Materials

The following supporting information can be downloaded at: https://www.mdpi.com/article/10.3390/cancers18040588/s1, Table S1: Descriptive comparison of rehabilitation vs. non-rehabilitation undergoing patients with MM.
