# Supplementary material for: Inpatient Rehabilitation Improves Physical and Mental Health in Multiple Myeloma: A Prospective Cohort Study
Source: Cancers (Basel). 2026 Feb 11;18(4):588. doi: 10.3390/cancers18040588 (PMC12939557; doi:10.3390/cancers18040588)
Supplement: Supplementary file 1 [file cancers-18-00588-s001.zip › cancers-4120961-supplementary.pdf]

Supplement Table 1. Descriptive comparison of rehabilitation vs. non-rehabilitation undergoing patients with MM

Variables	Rehabilitation cohort (n=60)	Non-rehabilitation group (n=6)
	Median (range) / n (%)	Median (range) / n (%)
Median age at initial diagnosis [years]<br>Median age at T0 [years]	59 (32-71)<br>63 (37-74)	67 (59-73)<br>69 (60-75)
Gender: male / female	32 (53) / 28 (47)	4 (67) / 2 (33)
Ethnicity: caucasian / others	55 (92) / 5 (8)	6 (100)
Marital status: married / others	45 (75) / 15 (25)	5 (83) / 1 (17)
Employment: retired / working	30 (50) / 30 (50)	3 (50) / 3 (50)
Income: medium or high / low*	59 (98) / 1 (2)	5 83) / 1 (17)
MM / plasmacytoma, AL-amyloidosis or SMM	54 (90) / 6 (10)	6 (100) / 0 (0)
Type of MM: IgG / IgA / LC / IgM and others	44 (74) / 11 (18) / 2 (3) / 3 (5)	3 (50) / 2 (33) / 1 (17)
LC type: kappa / lambda / biclonal	41 (68) / 18 (30) / 1 (2)	3 (50) / 3 (50)
ISS: I / II / III	20 (33) / 26 (43) /14 (24)	1 (17) / 2 (33) / 3 (50)
R-ISS: I / II / III	12 (20) / 35 (58) /13 (22)	2 (33) / 3 (50) / 1 (17)
Osteolytic lesions: 0 - 2 / 3 or more	20 (33) / 40 (67)	2 (33) / 4 (67)
Current state of disease: CR + nCR / vgPR + PR	27 (45) / 33 (55)	6 (100) / 0 (0)
Induction treatment:<br>Daratumumab-VCD / VCD / other	10 (17) / 48 (80) / 2 (3)	3 (50) / 3 (50)
Stem cell transplantation performed:<br>ASCT / ASCT + allo-SCT / standard therapy	52 (87) / 5 (8) / 3 (5)	6 (100) / 0 (0) / 0 (0)
Median (range) hemoglobin (g/dl)	12.5 (8.4-15.2)	11.8 (9.9-12.7)
Median (range) CRP (mg/l)	3 (1.5-58.0)	1.2 (0.9-7.8)
Median (range) proBNP (pg/ml)	111.5 (50-1473)	148 (90-328)
Median (range) LDH (U/l)	203 (141-390)	213 (159-243)
HRQoL: PCS at T0 → T1	36 (11-62) → 42 (20-57)	33 (21-41) → 33 (21-41)
HRQoL: MCS at T0 → T1	51 (19-65) → 56 (35-67)	55 (44-62) → 55 (44-62)
R-MCI: fit patients at T0 → T1	31 (52) → 32 (53)	4 (67) → 4 (67)
Fatigue[1] T0 → T1 (yes)	11 (12) → 2 (4)	4 (67) → 4 (67)
Depression[2] T0 → T1 (yes)	23 (38) → 5 (8)	0 (0) → 0 (0)
Self-rated fitness T0 → T1 *	3.1 → 4.9	7.0 → 7.7
PA/sports performed T0 → T1	20 (33) → 59 (98)	2 (33) → 2 (33)

Definitions + abbreviations:
T0: time point zero, baseline; T1: end of rehabilitation; MM: multiple myeloma; AL-amyloidosis: amyloid light chain; SMM: smoldering multiple myeloma; LC - type: light chain type; ISS: international staging system; R-ISS: revised international staging system; CR: complete remission, nCR: almost complete remission, vgPR: very good partial response, PR: partial remission. Daratumumab-VCD: daratumumab-bortezomib-cyclophosphamide-dexamethasone; other: daratumumab alone + immunoglobulin infusion. ASCT: autologous stem cell transplantation, allo-SCT: allogeneic stem cell transplantation; CRP: C-reactive Protein; proBNP: pro Brain Natriuretic Peptide; LDH: Lactate-dehydrogenase; HRQoL: health-related quality of life; PCS: physical component summary; MCS: mental component summary; R-MCI: revised myeloma comorbidity index, PA: physical activity; * on a 10-point scale from 0 (worst) to 10 (best)
* Income: medium-high >1100 € / month, low < 1100 € / month
1: Fatigue assessed according to CTCAE
2: Depression according to Hamilton (HDS-17); PA: physical activity
